# Supplementary material for: Complexome profiling of the Chlamydomonas psb28 mutant reveals TEF5 as an early PSII assembly factor
Source: Plant Cell. 2025 Mar 17;37(6):koaf055. doi: 10.1093/plcell/koaf055 (PMC12164590; doi:10.1093/plcell/koaf055)
Supplement: koaf055_Supplementary_Data [file koaf055_supplementary_data.zip › psb28_interactive.zip!/plots/intensity/qualityPlots/peptides/NUOB16_Cre16.g664600.t1.2.html]

### Cre16.g664600.t1.2

Trivial name: NUOB16  
SeqCoverage [median]: 58.50  
Mass: 16.37 kDa  
  
 

| Sequence | MissedCleavage | Mass [Da] | wtIntensity | muIntensity | Score |
| --- | --- | --- | --- | --- | --- |
| DMPIVQDAPPPGGFPTIR | 0 | 1906.96 | 1156.04 | 0.00 | 49.46 |
| EREEEAKVMK | 2 | 1247.62 | 1120.58 | 2332.88 | 94.62 |
| FEADEILTVR | 0 | 1191.61 | 4515.44 | 8015.27 | 164.68 |
| IVHPVLQAEWDLR | 0 | 1574.86 | 687.77 | 0.00 | 53.25 |
| RFEADEILTVR | 1 | 1347.71 | 11924.88 | 19712.73 | 98.11 |
| SVKDMPIVQDAPPPGGFPTIR | 1 | 2221.16 | 1299.18 | 13143.43 | 69.78 |
| TEVLKR | 1 | 744.45 | 29085.65 | 35610.26 | 99.51 |
| VGESTTR | 0 | 748.37 | 851.38 | 5231.30 | 104.45 |
| WLAPLESWDKRPLV | 2 | 1708.93 | 979.11 | 0.00 | 59.90 |
|  |  |  | 51620.0 | 84045.9 |  |
